# Whole-genome sequence of bacteriocin-producer *Lactococcus garvieae* SJC17 isolated from São Jorge cheese

**DOI:** 10.1128/mra.00639-26

**Published:** 2026-09-30

**Authors:** Célia C. G. Silva, Sofia Silva, Márcia Coelho, Susana Ribeiro

**Affiliations:** 1 School of Agrarian and Environmental Sciences, University of the Azores, Angra do Heroísmo, Portugal; 2 Institute of Agricultural and Environmental Research and Technology (IITAA), University of the Azores, Angra do Heroísmo, Portugal; Nanchang University, Nanchang, Jiangxi, China

**Keywords:** lactic acid bacteria, *Lactococcus garvieae*, bacteriocins, cheese

## Abstract

A strain isolated from artisanal São Jorge cheese that produced bacteriocin-like inhibitory substances was identified as *Lactococcus garvieae*. The genome consists of a draft assembly of 2,068,957 bp with 38.34% GC content. Genomic analysis revealed genes related to garvieacin Q production, providing insight into the strain’s potential antimicrobial properties.

## ANNOUNCEMENT

*Lactococcus garvieae* is a facultatively anaerobic, gram-positive coccus that occurs as a natural component of the microbiota of various raw-milk dairy products, particularly artisanal cheeses (1–3). Although recognized primarily as an opportunistic pathogen in fish (4) and, occasionally, in humans (5), dairy-derived strains often lack typical virulence determinants and can contribute positively to cheese ripening and flavor development (6). *L. garvieae* SJC17 was previously shown to produce a bacteriocin-like inhibitory substance with potent activity against the foodborne pathogen *Listeria monocytogenes*, making it a promising candidate for bioprotective applications (7). To gain insight into the genetic basis of its antimicrobial activity and to assess its biosafety, the genome of this strain was sequenced.

The strain was isolated from a raw-milk São Jorge PDO cheese sample collected at the UNIQUEIJO cooperative on São Jorge Island, Azores, Portugal (latitude 38.70°N, longitude 28.20°W). Cheese curd (25 g) was homogenized in a stomacher (400 Circulator; Seward Medical, London, UK) for 2 min at 230 rpm, serially diluted in 0.1% (wt/vol) peptone water, and plated on M17 agar, followed by aerobic incubation at 30°C for 72 h. A single colony was picked and re-streaked at least three times to obtain a pure culture. For identification, genomic DNA was extracted using the UltraClean Microbial DNA Isolation Kit (QIAGEN, Hilden, Germany) according to the manufacturer’s protocol, and the isolate was identified as *Lactococcus garvieae* by 16S rRNA gene sequencing using universal primers 46F and 1409R under the thermocycling conditions as described by Pires et al. (8). BLASTn (v2.10.1) analysis against the GenBank database showed 100% sequence identity with the reference strain *L. garvieae* strain DSM 20684 (GenBank CP118950).

For whole-genome sequencing, the strain was cultured in MRS broth (Biokar Diagnostics, Allonne, France) at 30°C for 24 h, and DNA was extracted using the same extraction kit and protocol as described above. Libraries were constructed with the KAPA EvoPrep Kit (Roche Sequencing Solutions, Basel, Switzerland) and sequenced in the Illumina NovaSeq 6000, using 150 bp paired-end sequencing reads. The sequencing run produced a total of 9,661,720 sequence reads, corresponding to an estimated 700.5× genome coverage (Table 1).

**TABLE 1:** Genome characteristics of *Lactococcus garvieae* SJC17

Characteristics	Value
Total genome size (bp)	2,068,957
Number of contigs	31
N50 (bp)	194,835
L50	4
G + C content (%)	38.34
Sequencing coverage	700.5×
Number of protein-coding genes	2,094
Number of tRNA genes	46
Number of rRNA genes	5
Phenotypic resistance	Tetracycline
Antibiotic resistance genes	*tet(S)*
Virulence factors	*lepa*, *purB*, *clpP*, *SP_1193*, *leuS*

Raw reads were quality-assessed using FastQC v0.11.9 (9) and then quality-trimmed using CLC Genomics Workbench v12.0.3 (10) with the following parameters: ambiguity limit = 2 nucleotides, quality limit = 0.01, and minimum read length after trimming = 30  bp, and short reads were discarded. Trimmed reads were assembled *de novo* using the de Bruijn graph algorithm implemented in CLC Genomics Workbench v12.0.3, with length fraction = 0.80, similarity fraction = 0.80, and a minimum contig size of 500 bp. The final draft genome assembly consists of 31 contigs with an N50 of 194,835 bp and a total length of 2,068,957 bp. The G + C content is 38.34% (Table 1). Assembly quality was assessed with QUAST v5.0.2 (11), and completeness was estimated using CheckM v1.1.3 (12) utilizing the *Lactococcus garvieae* marker set on the PGAP-generated gene set. The analysis resolved a genome completeness of 96.8% and a contamination level of 1.27%.

Genome annotation was performed using the Bacterial and Viral Bioinformatics Resource Center (BV-BRC 3.57.31, https://www.bv-brc.org), which predicted 2,094 protein-coding sequences, 46 tRNA genes, and 5 rRNA genes. The antibiotic resistance gene *tet*(S) was detected (Table 1), and phenotypic resistance to tetracycline was confirmed by disk diffusion on Mueller-Hinton agar following CLSI guidelines (13). While the presence of a tetracycline resistance gene is notable for a strain intended for food applications, it is important to note that tetracycline resistance is common among dairy-associated *L. garvieae* strains and does not preclude its use as a bioprotective culture (14).

Using the VirulenceFinder v2.0.5 database (15), we did not detect genes associated with Shiga toxin, pathogenic *Escherichia coli*, *Listeria* spp., *Enterococcus* spp., and *Staphylococcus aureus* toxins (https://cge.food.dtu.dk/services/VirulenceFinder/). The BV-BRC annotation pipeline identified the presence of general stress response and housekeeping genes (*lepa*, *purB*, *clpP*, *SP_1193*, and *leuS*) that are not considered specific virulence determinants in *L. garvieae*. Furthermore, the PathogenFinder v1.1 tool (https://cge.food.dtu.dk/services/PathogenFinder/) classified the strain as a non-human pathogen (16). All tools were used with default parameters unless otherwise stated. Preliminary analysis with BAGEL4 (17) identified a gene cluster encoding two putative class II bacteriocins of the garvieacin family (garvieacin Q), consistent with the strain’s known anti-*Listeria* activity.

## Data Availability

The draft genome assembly of *Lactococcus garvieae* SJC17 has been deposited at DDBJ/ENA/GenBank under accession number JCCAHB000000000. The version described in this paper is version JCCAHB010000000. The associated BioProject and BioSample identifiers are PRJNA1509978 and SAMN62282148, respectively. The raw sequencing data have been deposited in the NCBI Sequence Read Archive under accession number SRR40084272. The 16S rRNA gene sequence has been deposited in GenBank under accession number MT742851.
